# Einstellungen und Hinderungsgründe bezüglich Telemedizin bei Epilepsien: Eine Umfrage in neurologischen Praxen

**DOI:** 10.1007/s10309-021-00417-0

**Published:** 2021-06-30

**Authors:** Johann Philipp Zöllner, Anna H. Noda, Jeannie McCoy, Christian Roth, Doris Fischer, Edgar Bollensen, Karl-Heinz Henn, Laurent M. Willems, Anne-Christine Leyer, Susanne Schubert-Bast, Felix Rosenow, Adam Strzelczyk

**Affiliations:** 1 Epilepsiezentrum Frankfurt Rhein-Main, Zentrum der Neurologie und Neurochirurgie, Universitätsklinikum Frankfurt – Goethe-Universität Frankfurt, Schleusenweg 2–16 (Haus 95), 60528 Frankfurt am Main, Deutschland; 2 LOEWE Center for Personalized Translational Epilepsy Research (CePTER), Goethe-Universität Frankfurt, Frankfurt am Main, Deutschland; 3 Klinik für Neurologie, DRK Kliniken Nordhessen, Kassel, Deutschland; 4 Klinik für Neurologie, Universitätsklinikum Gießen und Marburg, Philipps-Universität Marburg, Marburg (Lahn), Deutschland; 5 Klinik für Pädiatrie, St. Vincenz-Krankenhaus, Limburg, Deutschland; 6 Praxis für Neurologie, Eschwege, Deutschland; 7 Klinik für Neurologie, Sana Klinikum Offenbach, Offenbach, Deutschland; 8 Klinik für Pädiatrie und Neuropädiatrie, Universitätsklinikum Frankfurt – Goethe-Universität Frankfurt, Frankfurt am Main, Deutschland

**Keywords:** Digitalisierung, Telekonsil, Epileptischer Anfall, Tele-EEG, Ländlicher Raum, Digitalization, Tele-consultation, Epileptic seizure, Tele-EEG, Rural areas

## Abstract

**Hintergrund:**

In Anbetracht ihres bedeutenden Potenzials zur Verbesserung der medizinischen Versorgung wird Telemedizin weiterhin zu wenig genutzt. Trotz einiger erfolgreicher Pilotprojekte in den vergangenen Jahren ist insbesondere über die Hindernisse der Etablierung und Verstetigung von Telemedizin wenig bekannt. Diese Studie hatte das Ziel, die Einstellung niedergelassener Neurologen hinsichtlich der Nutzung von Telemedizin in der Epileptologie und resultierende Hinderungsgründe zu verstehen. Gleichzeitig werden mögliche Lösungsansätze präsentiert.

**Methoden:**

Mithilfe eines individuell erstellten 14-Item-Fragebogens befragten wir prospektiv alle Neurologen, die zuvor die Teilnahme an einem transregionalen Telemedizinpilotprojekt im Bereich der Epileptologie abgelehnt oder keine Rückmeldung gegeben hatten, zu Gründen für und gegen den generellen Einsatz von bzw. die Teilnahme an Telemedizin.

**Ergebnisse:**

Von 58 kontaktierten Neurologen antworteten 33 (57 %). Die häufigsten Gründe für die fehlende Nutzung der Telemedizin waren ein vermuteter Zeitmangel oder ein vermuteter zu großer organisatorischer Aufwand (49 %). Zudem wurden Bedenken bezüglich der technischen Ausstattung (30 %) und eine Präferenz für alternative Wege der intersektoralen Kommunikation (30 %) angegeben. Befürchtete Probleme in Bezug auf die Kostenerstattung für telemedizinische Leistungen waren für 27 % ein Hindernis. Neurologen in ländlichen Gebieten waren signifikant häufiger bereit, zunächst eine telemedizinische Konsultation anzufordern, bevor sie eine Überweisung ausstellen (*p* = 0,006).

**Schlussfolgerungen:**

Die flächendeckende Etablierung von Telemedizinstrukturen ist immer noch durch Hindernisse erschwert, die meist im organisatorischen Bereich liegen. Die bestehenden Herausforderungen im Gesundheitswesen in ländlichen Gebieten sind eine besondere Chance für die Implementierung von Telemedizin. Die meisten Probleme der Telemedizin können gelöst werden, sollten aber bereits bei der Konzeptionierung von Projekten mitbedacht werden, um ihre Verstetigung zu erleichtern.

## Abstract (en)

**Background:**

Considering its significant potential for improving medical care, telemedicine continues to be underutilized. Despite some successful pilot projects in recent years, little is known about the barriers to the establishment and permanence of telemedicine. We conducted this study to understand the attitudes of neurologists in private practice regarding the use of telemedicine in epileptology and resulting barriers. At the same time, we present possible solutions.

**Methods:**

Using an individually designed 14-item questionnaire, we prospectively surveyed all neurologists who had previously not responded or had declined participation in a transregional telemedicine pilot project regarding perceived barriers to the use of telemedicine.

**Results:**

Of 58 contacted neurologists, 33 (57%) responded. The most common reasons for not using telemedicine were a perceived lack of time or a suspected excessive organizational burden (49%), concerns about technical equipment (30%), and a preference for alternative ways of intersectoral communication (30%). Feared problems related to reimbursement for telemedicine services were an obstacle for 27%. Neurologists in rural areas were significantly more willing to first request a telemedicine consultation before making a referral (*p* = 0.006).

**Conclusion:**

The widespread establishment of telemedicine is still hindered by obstacles, most of which are organizational. The existing healthcare challenges in rural areas are a particular opportunity for the implementation of telemedicine. Most barriers to telemedicine can be solved but should be considered from the onset when designing pilot projects in order to facilitate their continuation.

## Hintergrund und Fragestellung

Telemedizin bedeutetet die Erbringung einer medizinischen Leistung über Distanz unter Einsatz von Informations- und Kommunikationstechnologien (IT) [2]. Sie bietet eine Chance zum Ausgleich von Unterversorgung im ländlichen Bereich und kann die medizinische Versorgung insgesamt verbessern [3]. Ihr wird daher seit Anfang der 1990er-Jahre immer wieder der Durchbruch vorausgesagt [1, 10]. Es verwundert daher, dass sich dieser erst über den Umweg der klinischen Notwendigkeiten und Improvisation während der „Severe acute respiratory syndrome coronavirus type 2“(SARS-CoV‑2)-Pandemie seit dem Jahr 2020 abzeichnet [19].

In der Historie findet sich auch kein Mangel an Pilotprojekten; vielmehr zeigt sich, dass vielen Unternehmungen der Sprung in die Regelversorgung nicht gelang [1, 3]. Die möglichen Gründe sind dabei mannigfaltig; häufig wurden Finanzierung, Technik, Organisation, Akzeptanz und Motivation identifiziert [1, 3–6, 8, 13, 18]. Speziell in Deutschland werden pragmatische Lösungen durch die intersektorale Abschottung und teils wirklichkeitsferne Datenschutzanforderungen erschwert [3]. Hinzu kommen Defizite in der IT-Kompetenz von Ärztinnen, Ärzten und Pflegepersonal [3].

Als Haupthindernisse der Verstetigung der Telemedizin werden häufig Finanzierung und Technik angesehen. Dies sind jedoch nur die augenfälligsten Probleme, es lassen sich schließlich im deutschen Gesundheitssystem ausreichend Gegenbeispiele finden [9]. Ein wesentlicheres Hindernis ist vielmehr organisatorischer Natur [15]. Jedes Telemedizinsystem verändert als „virtuelle Organisation“ die Prozesse bei allen Teilnehmern [1]. Die sich daraus ergebenden administrativen Konsequenzen werden häufig unterschätzt [1]. Wie am Beispiel der Akzeleration der Telemedizin durch die SARS-CoV-2-Pandemie sichtbar wird, motiviert zur Nutzung von Telemedizin primär der (wahrgenommene) Bedarf und sekundär die grundsätzliche technische Machbarkeit [17, 18]. Weitere Problemfelder sind eine fehlende Standardisierung [3] und fehlender staatlicher Steuerungswille, der zur ineffizienten Konkurrenzsituation privatwirtschaftlicher E‑Health-Unternehmen auf dem Gesundheitsmarkt führt.

Die meisten bisherigen Studien sind Strukturanalysen einzelner Projekte [4]. Die Perspektive der potenziellen Anwender wird meist nur indirekt einbezogen. In einer fast 20 Jahre alten norwegischen Studie nannten Hausärzte als Hemmnisse v. a. zusätzlichen Zeitbedarf, fehlende Vergütung und eigene Unsicherheit in der Anwendung [8]. Die meisten in Deutschland evaluierten Teleneurologieprojekte befassen sich mit der Akutversorgung des Hirninfarktes [10]. Es bestehen weniger Erfahrungen in der Teleepileptologie.

Vor diesem Hintergrund befragten wir niedergelassene Fachärztinnen und Fachärzte für Neurologie (Neurologen), die an einem sektorenübergreifenden Telemedizinprojekt (EpilepsieNetz Hessen Evaluation [ENHE]) nicht teilnehmen wollten. Wir analysieren die häufigsten subjektiven Hinderungsgründe und zeigen auf, wie im ENHE die potenziellen Hemmnisse der Telemedizin mitbedacht werden.

## Studiendesign und Untersuchungsmethoden

Das ENHE (https://portal.epilepsienetz-hessen.de/) ist ein Arzt-zu-Arzt-Telemedizinsystem, über welches Neurologen Konsile zu epileptologischen Fragestellungen an spezialisierte Epileptologen in Epilepsiezentren in Hessen richten können. Über das ENHE können alle zur Befundung relevanten Daten (Elektroenzephalogramm [EEG], Bildgebung, schriftliche Befunde) im Originalformat übertragen werden, sodass eine Befundung entsprechend Empfehlungen der Deutschen Gesellschaft für Klinische Neurophysiologie und Funktionelle Bildgebung (DGKN) möglich ist [7]. Die Teilnahme ist im Projektzeitraum für alle neurologischen Zuweiser kostenlos. Das ENHE wird durch das Hessische Ministerium für Soziales und Integration (HMSI) und das Hessische Ministerium für Wissenschaft und Kunst (HMWK) finanziert.

Wir schlossen niedergelassene Neurologen aus Hessen in die Studie ein, denen zuvor die Teilnahme an dem ENHE angeboten worden war. Die Ärzte hatten die Teilnahme an dem Projekt explizit abgelehnt oder auf mehrfache Einladung hin kein Interesse bekundet. Diese Ärztinnen und Ärzte wurden im Herbst 2020 standardisiert mittels eines für diese Umfrage erstellten Fragebogens befragt. Der Fragebogen enthielt 14 Fragen in 6 Kategorien zu organisatorischen, technischen, finanziellen, patientenbezogenen und ideologischen sowie projektspezifischen Aspekten (s. Tab. 1). Wir formulierten die Fragen auf der Grundlage bekannter allgemeiner und spezieller Hemmnisse der Telemedizin [3, 6, 13, 18]. Mehrfachantworten waren möglich. Die Ländlichkeit des Praxisstandortes ermittelten wir auf Basis des Thünen-Atlas [16]. Die ausschließlich nominalen Ergebnisse werden als Anzahl mit Frequenz berichtet. Wir verglichen zudem die einzelnen Antworten hinsichtlich der Ländlichkeit des Praxisstandortes mittels zweiseitiger Fischer Exakt-Tests; ein *p*-Wert von < 0,05 (zweiseitig) wurde als Grenze statistischer Signifikanz angesehen.

**Table 1:** 

Einer Teilnahme am EpilepsieNetz Hessen Evaluation (ENHE) haben folgende Punkte entgegengestanden: *(Bitte ankreuzen, Mehrfachnennungen möglich)*	
1. Die Anbindung an das Epilepsienetz ist zu aufwendig: *(Bitte ankreuzen, Mehrfachnennungen möglich)*	
☐	Vertragsprüfung zu aufwendig
☐	Technische Anbindung zu aufwendig
☐	Aufklärung der Patienten und Fragebögen der Begleiterhebung zu aufwendig
☐	Konsilstellung zu zeitaufwendig
☐	Mir fehlt die Zeit, mich mit dem Projekt/den Unterlagen/der Anbindung an das EpilepsieNetz Hessen auseinanderzusetzen
2. Meine technische Ausstattung steht einer Teilnahme entgegen: *(Bitte ankreuzen, Mehrfachnennungen möglich)*	
☐	Keine Netzwerkverbindung in der Praxis
☐	Keine Netzwerkverbindung am EEG-Computer
☐	Hardware nicht vorhanden/nicht leistungsfähig genug
☐	Betriebssystem nicht kompatibel
3. Keine ausreichende technische Expertise in der Praxis	
4. Die Anbindung an das EpilepsieNetz Hessen ist mit zu hohen Kosten verbunden (z. B. durch IT-Arbeitsstunden, Anschaffung neuer Hard‑/Software), falls ja, geschätzte Summe/Kostenvoranschlag: __________	
5. Keine relevante Anzahl an Epilepsiepatienten in Behandlung	
6. Meine Epilepsiepatienten mit komplexer Behandlung sind bereits an ein Epilepsiezentrum angebunden	
7. Ich überweise meine Epilepsiepatienten mit komplexer Behandlung lieber, als dass ich ein Konsil stelle	
8. Meine Epilepsiepatienten mit komplexer Behandlung bevorzugen es, sich persönlich am Epilepsiezentrum vorzustellen	
9. Ich bin von dem Konzept des EpilepsieNetz Hessen nicht überzeugt	
10. Ich stehe der Telemedizin im Allgemeinen kritisch gegenüber	
11. Die unzureichende Vergütung von telemedizinischen Leistungen ist ein Problem	
12. An einem Pilotprojekt möchte ich nicht teilnehmen, wäre einer telemedizinischen Regelversorgung gegenüber aber offen	
13. Ich hätte gerne mehr Informationen über das Projekt erhalten	
14. Ich möchte noch folgende Anmerkungen machen: __________	

## Ergebnisse

### Rücklaufquote und Teilnehmer

Von den 58 angefragten Neurologen füllten 33 den Fragebogen aus und sendeten ihn zurück (Rücklaufquote 57 %). Von diesen waren 12 in sehr ländlichen, 10 in eher ländlichen und 11 in nichtländlichen Regionen tätig.

### Hinderungsgründe nach Bereichen

**Figure 1:**
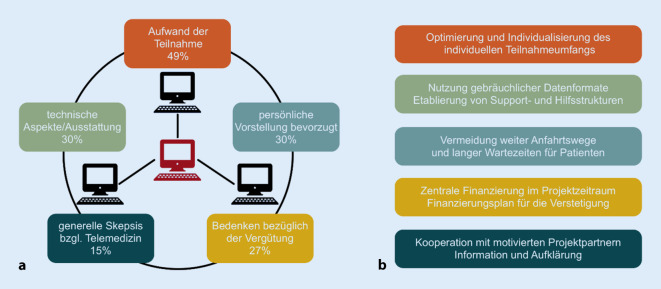

- *Organisation:* Der am häufigsten genannte Hinderungsgrund war, die Anbindung an das Telemedizinprojekt sei zeitlich und/oder organisatorisch zu aufwendig (*n* = 16, 49 %) (Abb. 1). Speziell genannt wurden Zeitmangel, sich mit dem Projekt auseinanderzusetzen (*n* = 14, 42 %), der Aufwand zur Einrichtung der Technik (*n* = 5, 15 %), bei der Vertragsgestaltung (*n* = 3, 9 %) und bei der Erhebung der wissenschaftlichen Begleituntersuchung (*n* = 3, 9 %). Nur einer der Befragten fand die Konsilstellung an sich zu aufwendig.
- *Technik:* Hier gaben 10 (30 %) Neurologen an, dass die technische Ausstattung der Praxis einer Teilnahme grundsätzlich entgegenstehe. Obwohl alle Praxen über eine Internetverbindung verfügten, bestand bei 10 (30 %) keine Netzwerkverbindung zum EEG-Gerät. Insgesamt 3‑mal (9 %) wurde bestehende Hardware und 2‑mal (6 %) Software als technisches Hemmnis genannt, 3 Neurologen (9 %) berichteten, es fehle insgesamt an technischer Expertise.
- *Arzt- und Patientenpräferenz:* Es gaben 10 Neurologen (30 %) an, Patienten lieber persönlich oder per Überweisung in einem Epilepsiezentrum vorzustellen. Insgesamt 10-mal (30 %) wurde berichtet, alle infrage kommenden Patienten seien bereits an einem Epilepsiezentrum angebunden. Neun Neurologen (27 %) erwarteten auch eine Patientenpräferenz für eine persönliche Vorstellung am Epilepsiezentrum.
- *Kosten/Vergütung:* Insgesamt 9 Neurologen (27 %) erwarteten Probleme bei der Vergütung telemedizinischer Leistungen. Spezifisch zu hohe Kosten für eine Teilnahme am ENHE erwarteten 4 Neurologen (12 %). Dabei wurden mit der Teilnahme verbundene Kosten zwischen 1000 und 30.000 € erwartet bzw. vermutet. Ein Neurologe äußerte Sorge bezüglich nicht abschätzbarer Kosten.
- *Grundlegende Bedenken* gegenüber der Telemedizin im Allgemeinen äußerten 5 (15 %) der Neurologen.
- *Spezifische Bedenken *gegenüber dem Konzept des ENHE äußerten 3 (9 %) Neurologen. Insgesamt 6 Neurologen (18 %) wären nur an der Teilnahme an einem Telemedizinprojekt der Regelversorgung interessiert.

### Faktor Ländlichkeit

Bei der Beantwortung der Fragen zwischen unterschiedlich ländlich gelegenen Praxen ergab sich nur ein signifikanter Unterschied: Ärzte mit Praxen im nichtländlichen Raum gaben signifikant häufiger an, Patienten direkt zu überweisen statt zuvor ein Telekonsil stellen zu wollen (*p* = 0,006).

## Diskussion

In der vorliegenden Studie untersuchten wir Hinderungsgründe für die Teilnahme an Telemedizinprojekten, exemplifiziert an einem Arzt-zu-Arzt-Telemedizinprojekt in der Epileptologie (ENHE). Als wichtigste Gründe fanden wir einen vermuteten Zeitmangel bzw. zu hohen vermuteten Aufwand hinsichtlich der Telemedizin, Sorgen bezüglich der technischen Ausstattung und die Präferenz alternativer Wege der intersektoralen Kommunikation. Dabei berichteten v. a. Ärzte in nichtländlichen Praxen, die direkte Überweisung des Patienten einem Telekonsil vorzuziehen. Dies ist wahrscheinlich darauf zurückzuführen, dass Ärzte im städtischen Raum die Hürden für den Patienten, einen weiteren Termin an einem Zentrum wahrzunehmen, als geringer einschätzen.

Interessanterweise decken sich die häufigsten Problembereiche fast vollständig mit einer fast 20 Jahre alten Studie aus Nord-Norwegen [8]. Trotz der zeitlichen Distanz zu dieser Studie, der unterschiedlichen Adressaten (Hausärzte vs. Neurologen) und der sehr unterschiedlichen Bevölkerungsdichte in den untersuchten Regionen sind die Problembereiche fast identisch. Dies unterstützt die Annahme, dass die relevanten Probleme weniger in der konkreten Umsetzung, sondern eher in (überdauernden) organisatorischen Schwierigkeiten zu suchen sind [1, 15].

Aufschlussreich ist vor diesem Hintergrund die von fast einem Drittel der ablehnenden Neurologen geäußerte Präferenz konventioneller intersektoraler Kommunikation, v. a. über den Weg der Überweisung. Diese ist zwar für den Überweisenden nur wenig aufwendig, führt jedoch durch den Informationsverlust zu Zusatzaufwand. Gleichzeitig zeigt dies den wichtigen motivationalen Aspekt der Telemedizin. Wenn die Vorteile der telemedizinischen Kommunikation (fehlende Medienbrüche, Zeitgewinn für den Patienten, Informationsgewinn) nicht erkannt oder als relevant angesehen werden, ist eine Nutzung unwahrscheinlich. Auf der anderen Seite zeigt sich das Potenzial der Telemedizin gerade in der Versorgung des ländlichen Raumes, in dem Ärzte die Überweisung eines Patienten signifikant häufiger als „erste Wahl“ ansehen. Hier sind die Hürden eines zusätzlichen Arztbesuches für die oft wenig mobilen Epilepsiepatienten deutlich höher [20], und somit ist auch die Motivation, sich auf neue digitale Kommunikationswege einzulassen, größer.

Unsere Erkenntnisse liefern gleichfalls Ansätze für den möglichen Erfolg eines Telemedizinprojektes. Ein wesentlicher Faktor ist die Planung eines Projektes entlang eines nicht gedeckten Versorgungsbedarfes. Dies kann neben der bereits erwähnten räumlichen Lücke auch ein Engpass an spezieller Expertise, z. B. in der Neuropädiatrie, sein. Bereits bei der Planung des ENHE wurde der ländliche Raum als Zielbereich mitbedacht. Schon bei Beginn des Projektes sollten die Erwartungen der Teilnehmer abgefragt werden, auch konkrete Fragen nach der aufwendbaren Zeit und Ressourcen sind dabei wichtig. Es empfiehlt sich, in der Initialphase auf eine geringere Zahl an hoch motivierten Partnern zu setzen, da sich zu Beginn eines Projektes Strukturen und Abläufe noch ändern und Probleme gelöst werden müssen, die weniger motivierte Partner vom Projekt abspringen lassen. Hier ist die Identifizierung von Partnern mit einer Kombination aus hoher intrinsischer Motivation, hohem klinischem Bedarf mit gleichzeitiger Offenheit für Kooperationen wichtig [4]. Eine gut erreichbare und lösungsorientierte Supportinfrastruktur auf organisatorischer und technischer Ebene ist in dieser Phase besonders wichtig [4].

Da die technischen Gegebenheiten der Teilnehmenden gerade im niedergelassenen Bereich praktisch nicht innerhalb eines üblichen Projektzeitraumes geändert werden können, sollten von Anfang an offene technische Standards gewählt werden und bei Bedarf eine Anpassungsmöglichkeit der Infrastruktur möglich sein. Zudem sollte eine bessere Anwenderfreundlichkeit der EEG-Systeme von den Herstellern gefordert werden [22]. Zum Beispiel wird im Bereich der Epileptologie durch die Möglichkeit der Speicherung des EEG im Digital Imaging and Communications in Medicine Standard (DICOM) in Kürze eine Lösung für die bislang vorherrschende Inkompatibilität proprietärer EEG-Formate entstehen [12, 23]. Das ENHE bietet dabei die Möglichkeit des DICOM-Transfers. Dabei sind parallele Kommunikationskanäle zwar zu vermeiden, aber ggf. notwendig, falls intersektorale Grenzen sonst nicht überwunden werden können.

Finanzielle Hürden der Telemedizin werden sich wohl erst lösen lassen, wenn Anwendungen in ausreichender Breite ihre Wirtschaftlichkeit bewiesen haben. Die rechtlichen Rahmenbedingungen für die Abrechnung telemedizinischer Leistungen entstehen zwar derzeit, die Gebührensätze decken jedoch nicht die in Pilotprojekten entstehenden Entwicklungskosten ab, die nachfolgend der Regelversorgung zugutekommen können. Eine weitere staatliche Förderung von Pilotprojekten ist sinnvoll, um einen Abbruch von bereits begonnenen Projekten und damit den Verlust bereits aufgebauter Kompetenz zu vermeiden. Eine Unterbrechung eines Telemedizinnetzes für nur wenige Monate kann die aufgebauten Strukturen langfristig stören.

Limitationen der Planbarkeit von meist mehrjährigen Telemedizinprojekten ergeben sich aus der Dynamik der legislativen als auch organisatorischen Veränderungen innerhalb eines Projektes. Problematisch ist dabei z. B. der Wegfall des Ansprechpartners innerhalb einer teilnehmenden Organisation/Praxis, wenn für die Teilnahme v. a. ein Mitarbeiter zuständig war. Dies spricht für eine geringe Integration der „virtuellen Organisation“ Telemedizin in die lokale Struktur. Von Anfang an sollten mehrere Ansprechpartner bei jeder teilnehmenden Organisation ausgewählt werden. Der Einfluss des Projektführers auf die Teilnehmer ist dabei natürlicherweise begrenzt, die Wichtigkeit der lokalen Verankerung mit Benennung mehrerer Ansprechpartner sollte jedoch deutlich gemacht werden. Die Einführung von Standard Operating Procedures (SOPs) kann bei der Integration helfen.

Zusätzlich könnten sich Probleme der Telemedizin auf Patientenebene (z. B. eine fehlende Akzeptanz) zeigen; diese Problemebene wird in der vorliegenden Arbeit nicht berücksichtigt.

Ein wichtiger Faktor für die mittelfristige wissenschaftliche und ökonomische Anerkennung der Telemedizin ist ihre Evaluation. Obwohl Daten aus der Tele-Stroke-Versorgung existieren [6, 10], besteht in anderen Teilen der Teleneurologie nur eine geringe Evidenz. Deshalb beinhaltet das ENHE eine zwingende versorgungsforscherische Begleituntersuchung [11, 14, 21], die die Akzeptanz des Projektes systematisch untersucht und so eine Argumentationsgrundlage für die weitere Verbreitung der Teleepileptologie darstellen kann.

## Fazit für die Praxis

Wesentliche Hinderungsgründe für die Umsetzung und Etablierung von Telemedizinprojekten sind ein vermuteter zu hoher organisatorischer Aufwand, technische Hürden und Bedenken vor zu hohen Kosten. Dabei sind Ärzte im ländlichen Bereich eher bereit, Telemedizin zu nutzen und damit konventionelle Überweisungen von Patienten zu ersetzen. Wie im ENHE geschehen, sollten der potenzielle Nutzerkreis, ein relativ offener technischer und adaptierbarer Standard sowie ein engmaschiger Support gerade der Pilotnutzer mitbedacht werden. Eine Förderung auf Landesebene und durch die Kostenträger kann die Überführung der erworbenen telemedizinischen Kompetenzen in die Regelversorgung sicherstellen. Neben der Vergütung der ärztlichen Leistung sind auch die erheblichen Kosten für Entwicklung und Etablierung zu bedenken.
